# Morphological and Physical Profile of a Collegiate Water Skier

**DOI:** 10.3390/ijerph18031150

**Published:** 2021-01-28

**Authors:** Michael A. Woodgate, Joshua J. Gann, William Hey, Hyun Chul Jung

**Affiliations:** 1 Department of Kinesiology, University of Louisiana at Monroe, 700 University Ave Monroe, Monroe, LA 71209, USA; woodgate@ulm.edu (M.A.W.); gann@ulm.edu (J.J.G.); hey@ulm.edu (W.H.); 2 Department of Coaching, College of Physical Education, Global Campus, Kyung Hee University, 1732 Deokyoungdaero, Giheung-gu, Yongin-si, Gyeonggi-do 17014, Korea

**Keywords:** somatotype, fitness testing, water ski performance, body composition, collegiate athletes

## Abstract

This study aimed to examine morphological and physical fitness profile in collegiate water skiers and to identify the potential morphological and physical fitness factors, important for success in the slalom, trick, and jump events. Twenty collegiate water skiers were subject to anthropometric, somatotype measurements and a battery of physical tests inclusive of water ski-specific fitness variables. An independent *t*-test was used to compare the gender differences of dependent variables. Partial correlation and linear regression analyses were used to identify the factors that are associated with water ski performance. Male water skiers were lower in endomorphic component and better in power, speed, and cardiorespiratory fitness than female water skiers (*p* < 0.05). Somatotype such as mesomorphic (r = −0.48) and ectomorphic components (r = −0.60), sum of hand-grip strength (r = 0.98), and muscular endurance including posterior extension (r = 0.59) and left lateral flexion (r = 0.63) were significantly correlated with water skiing performance score (*p* < 0.05). The results of regression analyses showed that mesomorphic component (r^2^ = 0.24, *p* = 0.04), sum of hand-grip strength (r^2^ = 0.95, *p* = 0.001), and muscular endurance (r^2^ = 0.30, *p* = 0.03), appear to be crucial factors associated with water ski performance in slalom, trick (hands pass), and the jump events, respectively. Our study suggests that different morphological and fitness components are required to succeed in each tournament water skiing event. Coaches and athletes can utilize the battery of physical tests and design a specialized training regimen for each tournament water skiing event.

## 1. Introduction

Tournament water skiing is characterized by the three disciplines of slalom, tricks, and jumps. Participants can ski in either one, two, or all three events. The slalom event requires an athlete to navigate a course of six equidistant buoys. The boat speed is increased in predetermined increments, after each pass, up until the maximum speed of 36 mph for men and 34.2 mph for women, then the towline is incrementally shortened after each pass until a skier fails to make it successfully around a turn buoy [1]. The trick event is composed of two 20-s passes (1 × 20-s pass, collegiately) where a skier performs a series of surface and wake tricks, inclusive of rotations, flips, ski-line tricks, and toe-hold maneuvers, which are scored by a panel of judges [2]. The jump event is completed on a pair of skis, where the skier navigates through a course, building speed before propelling into a fixed ramp (5 feet collegiately, 6 feet professionally) and aiming to jump as far as possible, land, and ski away [3].

Each event in tournament water skiing requires different morphological and physiological capacities, in order to excel. The anthropometric features of athletes have been shown to play an important role in talent identification and performance criteria in sports [4]. Particularly, the understanding of morphological characteristics such as somatotype supports the impact of body shape on performance. Individual somatotype is demonstrated by the three numbered rating that represents endomorphic, mesomorphic, and ectomorphic components, and is simply categorized to seven somatotypes including central, endomorph, endomorph-mesomorph, mesomorph, mesomorph-ectomorph, ectomorph, ectomorph-endomorph [4]. Typically, elite gymnasts were identified as ectomorph-mesomorph [5] or balanced mesomorph while professional junior surfers were identified as ectomorph-mesomorph [6]. Different somatotype has also been identified by playing position in team sports such as soccer and basketball [7,8], and associated with aerobic, anaerobic, and overall sports performance [9]. Although various predicted models have been introduced to identify the anthropometric and physical fitness factors for success in athletic performance, no study existed with water ski performance.

Water skiing is categorized as an extreme sport and it requires significant physical capacities to excel in performance. Various physical and physiological factors have been examined in the previous studies and the authors reported that dynamic balance, muscular strength, and endurance are the important factors that contribute to water-skiing performance. Particularly, sustained isometric muscular actions are required to a maintain balanced posture on the dynamic water surface.

Currently, limited research literature exists regarding water skiing. The majority of available literature focuses on water ski-related injury [2,3,10]. Previous research into physiology in water skiing has been conducted [11,12,13,14] however, no previous research has showed the understanding of morphological and physical fitness profile of male and female water skiers. Additionally, it is important to understand the factors that are associated with water ski performance. Therefore, this study aimed (1) to examine morphological and physical fitness profile in collegiate water skiers and (2) to identify potential morphological and physical fitness factors, important for success in the slalom, trick, and jump events.

## 2. Materials and Methods

### 2.1. Participants

This is a cross-sectional study. A convenience sampling method was applied where 20 collegiate male and female water skiers, aged 18–25 years old, from the USA National Championship winning Water Ski Team voluntarily participated in the study. An explanation of the study’s purpose, procedures, potential risks, and benefits were presented orally by the investigator. Each participant completed a written informed consent approved by the Institutional Review Board at the University (IRB 892-2018). Participants also completed a written risk stratification screening form, and all reported no contraindications to exercise. Seated blood pressure and resting heart rate were taken, according to ACSM guidelines [15]. Participants were instructed to report to the Human Performance Laboratory, well hydrated and having abstained from alcohol and caffeine in the 24-h prior to their appointment. A recovery period of 3 min was given between each test and room temperature was controlled between 18 and 20 °C, with relative humidity maintained.

### 2.2. Assessments

#### 2.2.1. Anthropometric Measurements

Height and body weight were assessed on a stadiometer and balance scale (Detecto, Webb City, MO, USA). Percent body fat was estimated through the Jackson and Pollock 3-site skinfold method, males; chest, abdomen, mid-thigh, females; triceps, abdomen, mid-thigh with measurements taken using Lange calipers (Lange, Cambridge, MD, USA). Wingspan was measured, fingertip to fingertip, with participants extending arms laterally [16,17]. Additionally, anthropometric assessment including sitting height, leg length, body circumference at upper arm, flexed upper arm, waist, hip, mid-thigh, and mid-calf with a measuring tape, bone width at the distal of elbow and knee with small spreading caliper (GPM Anthropological Instruments 170, Zurich, Switzerland), and five sites of skin fold thickness (triceps, subscapular, suprailiac, thigh, and medial calf) were measured by the trained investigator to estimate the individual’s three-somatotype rating including ectomorphic, mesomorphic, and endomorphic components. The Heath–Carter anthropometric somatotype methods were applied to calculate each somatotype component [18]. Based on somatotype analysis, participants were classified to following seven categories; central, endomorph, endomorph-mesomorph, mesomorph, mesomorph-ectomorph, ectomorph, ectomorph-endomorph and individual result was plotted on a two-dimensional somatochart.

#### 2.2.2. The Battery of Physical Fitness Tests for a Water Skier

*Flexibility.* The flexibility test consisted of participants sitting with legs extended, feet flat against the measurement box. While keeping the legs flat against the floor, participants placed their hands on top of each other and reached forward as far as possible in a slow controlled movement. Three attempts were performed, with the highest measurement recorded [15].

*Y-Balance test.* The test required the participant to balance on one leg, whilst simultaneously reaching as far as possible with the other leg in three separate directions: anterior, posterolateral, and posteromedial. Participants completed three trials with each leg, with a composite reach distance determined from the sum of Y- measures/3- times the limb length × 100 [19].

*Explosive Power.* Vertical jump height was assessed using a Vertec height measurement system (Vertec, Sports Imports, Hilliard, OH, USA). Participants performed three countermovement jumps, and the highest attempt was recorded. A 1-min recovery period was given between each attempt.

*Speed and Agility Testing.* Participants performed a 5-min, self-selected, warm-up and familiarized themselves with proper use of timing equipment and testing protocol. Participants completed three, 30-m sprints, with each sprint followed by a 2-min recovery period. Following sprint testing, participants completed two trials of a *t*-test to assess agility. This test consisted of a sprint of 10 yards, shuffling to the right for five yards, shuffling back to the left for 10 yards, then back to the right for five yards, and then backpedaling 10 yards back to the start point. All sprint and agility times were assessed with an electronic timing system (Brower Timing Systems, Draper, UT, USA).

*Muscular Strength and Endurance.* Muscular strength was assessed via hand-grip dynamometer (Jamar, Bolingbrook, IL, USA). Participants held the dynamometer in their hand, with their arm extended anteriorly and then squeezed the dynamometer with maximum isometric effort for approximately 5 s. Participants performed three trials for each hand, alternating hands between each attempt. The score for the highest maximal isometric contraction for each hand was recorded. Muscular endurance was assessed by participants squeezing the dynamometer with maximal isometric effort until volitional fatigue or when the force of the squeeze dropped below 70% of the previously established max. Time to exhaustion was recorded with a manual stopwatch. Participants completed three trials with each hand, with a 2-min recovery period between each trial [20]. Core endurance and stability was measured using a previously established protocol [21]. This protocol consists of isometric contraction tests in three positions: trunk extended, right lateral flexion, and left lateral flexion. Participants held isometric contractions in each position until volitional fatigue. Time to exhaustion was measured with a manual stopwatch, and each trial separated by a minimum of 3 min. Participants completed as many push-ups [15], as a modified version of the ACSM push-up test of muscular endurance. Number of body weight squats possible in 1 min was also assayed, as adapted from the ACSM push up test. Participants were instructed on proper form for each movement and the number of each movements completed was recorded. Each test was separated by a minimum of 2 min.

*Cardiorespiratory Fitness.* Prior to testing, participants were fitted with a heart rate monitor (Polar Electro, Kempele, Finland). VO_2max_ was assessed via graded exercise test on a cycle ergometer. Metabolic data was collected using a metabolic cart (Quark CPET, Cosmed, Rome, Italy) and calibrated before each test using known gases. Bicycle seat height was adjusted to achieve a slight bend of the knee, with the pedal at the 6 o’clock position. Handlebars were also adjusted for personal preference. Each participant was fitted with a facemask and connected to the metabolic cart. As a warm-up, participants completed 3 min of pedaling (60 rev/min) with no resistance. After the warm-up, resistance was increased by 0.5 kilopond, each minute, until the participant reaches volitional exhaustion, cadence cannot be maintained, or investigators deemed it unsafe to continue. Heart rate was recorded every minute. Participants estimated their rating of perceived exertion (RPE) each minute using the Omni RPE scale [22].

*National Collegiate Water Ski Association Rankings System*. To identify the predictable factors of somatotype and physical fitness components for achieving optimal water ski performance, each skier’s best performance score of the season was taken from the National Collegiate Water Ski Association (NCWSA) Ranking’s list [23]. The score included individual best performance in each discipline at any eligible NCWSA event. In this study, 15 athletes were ranked in slalom, seven athletes in trick toes, six athletes in trick hands, and 13 athletes in jump from the NCWSA Ranking’s lists.

#### 2.2.3. Statistical Analysis

Data analysis was performed with SPSS version 23 (SPSS institute, Chicago, IL, USA). Descriptive statistics, including means, standard deviations, and 95% confidence interval (95% CI) were calculated for all anthropometrical and somatotype attributes, as well as all performance variables. An independent samples *t*-test for equality of means, with sex as the grouping variable, were performed to compare gender differences of anthropometric, somatotype, and physical fitness testing variables. Cohen’s d effect size was calculated to determine the mean difference between the groups, indicating small effect ≥ 0.2, medium effect ≥ 0.5, and large effect ≥ 0.8 [24]. Partial correlation analyses with sex as covariate were applied to examine the relationship between water ski performance and dependent variables. Simple and stepwise multiple linear regression analyses were applied using the significant variables from the linear correlation, to identify potential morphological and physical attributes for optimal water ski performance, in the slalom, trick, and jump events. The alpha level of statistical significance was set at ≤0.05.

## 3. Results

### 3.1. Anthropometric and Somatotype of Collegiate Water Skier

The results of anthropometric and somatotype variables are presented in Table 1. There were significant differences in body weight, height, body fat percentage, and sum of skinfold thickness, between males and females, respectively (*p* ≤ 0.05). Male water skier was significantly lower in endomorphic component than female water skier (*p* < 0.001), but no significant differences were observed in mesomorphic and ectomorphic components between groups.

**Figure 1:**
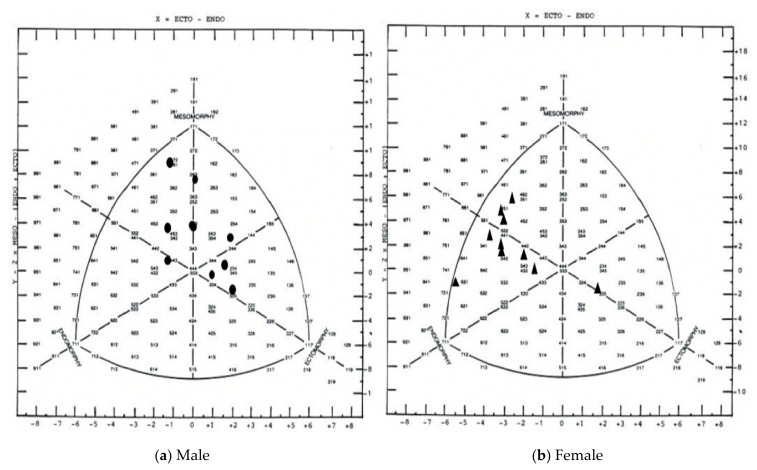
Somatochart of male and female water skiers.

Figure 1 shows a somatochart of the male and female water skiers. The male participants primarily represent as mesomorph-ectomorphs, mesomorphs, and endomorph-mesomorphs, with one participant exhibiting traits of an endomorph-mesomorph. However, revealed with the exception of one female who exhibited ectomorph traits, females primarily represent endomorph component, with mesomorph-endomorphs and endomorph-mesomorphs, comprising the rest of the somatochart.

### 3.2. Water Ski-Specific Physical Fitness

As shown is Table 2, significant differences between males and females in vertical jump, 40-yard dash, *t*-test, and VO_2max_ were observed (*p* ≤ 0.05). However, other fitness variables were not different between the groups.

### 3.3. Predictable Factors for Water Ski Performance

The results of partial correlation analysis with sex as a covariate are presented in Table 3. While a mesomorphic component had a significant negative correlation with the slalom ski performance (r = −0.48, *p* = 0.04), this somatotype component was positively correlated with toes performance (r = 0.83, *p* = 0.02). There was a strong positive correlation between sum of hand grip strength trick hands performance (r = 0.98, *p* = 0.001). Regarding ski jump, ectomorphic component (r = −0.60, *p* = 0.04), extension (r = 0.59, *p* = 0.05), and left lateral flexion (r = 0.63, *p* = 0.006) had a significant correlation with performance. Other physical fitness variables were not significantly correlated with water ski performance score.

**Figure 2:**
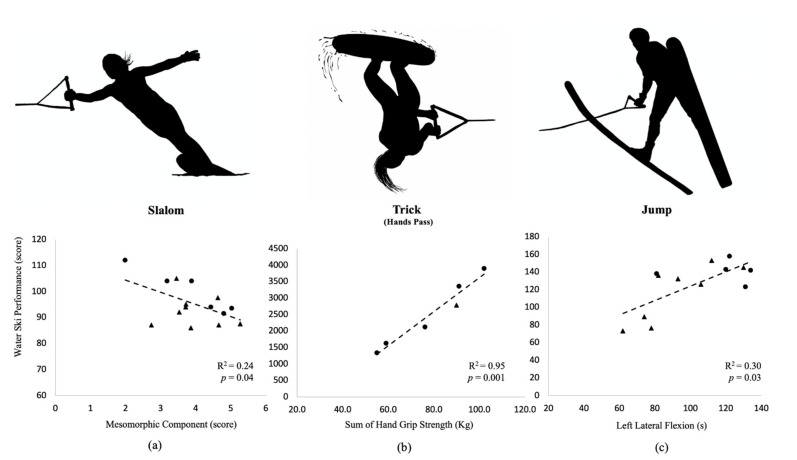
A regression analysis between selected factors and water ski performance in slalom, trick, and jump. Note: Circle represents a male water skier; triangle represents a female water skier. (**a**) a result of regression analysis between slalom performance score and mesomorphic component; (**b**) a result of regression analysis between trick hand pass performance score and sum of hand grip strength; (**c**) a result of regression analysis between jump performance score and left lateral flexion.

The regression analysis between slalom performance and the selected factor revealed that mesomorphic component had a significant contribution to the slalom performance with adjusted R^2^ = 0.24, F = 5.35, SEE = 6.79, *p* = 0.04, indicating that 24% of the variance of slalom performance can be accounted for by mesomorphic component (Figure 2a).

The regression analysis between trick (hands) performance and the selected factor revealed a sum of hand grip strength had a significant contribution to the trick (hands) performance with adjusted R^2^ = 0.95, F = 72.17, SEE = 256.78, *p* = 0.001, indicating that 95% of the variance of trick (hands) performance can be accounted for by sum of hand grip strength (Figure 2b).

The stepwise regression analysis between ski jump performance and the selected factors demonstrated muscular endurance (left lateral flexion) data was significantly contributed to the ski jump performance with adjusted R^2^ = 0.30, F = 6.45, SEE = 25.38, *p* = 0.03, indicating 30% of the variance of ski jump performance can be accounted for by left lateral flexion (Figure 2c).

## 4. Discussion

This study aimed to examine morphological and physical fitness profile in collegiate water skiers and to identify potential morphological and physical fitness factors, important for success in the slalom, trick, and jump events with reference to the athletes on the 2018 USA National Championship winning Water Ski Team. Findings indicate that male water skiers were lower in endomorphic component and better in performance variables such as vertical jump, 40-yard dash, *t*-test, and VO_2_ max, than female water skiers. A mesomorphic component was associated with slalom performance, while left lateral flexion was associated with jump performance. Especially, sum of hand-grip strength was strongly associated with trick (hands) performance.

### 4.1. Morphological and Physical Fitness Profile of Male and Female Water Skiers

In the present study, collegiate male water skiers represent as mesomorphic ectomorphs, mesomorphic mesomorphs, and endomorphic mesomorphs; similarly to adult professional basketball players [25] who exhibit quite a range throughout the somatochart, due to sport-specific positional tendencies. Somatotype for water ski athletes was also similar to sprint athletes, 2.1 ± 0.7, 5.0 ± 1.2, and 2.6 ± 0.9, for endomorphy, mesomorphy, and ectomorphy, respectively [26]. Figure 1 showed female participants to exhibit primarily endomorphic component, with mesomorphic endomorphs and endomorphic mesomorphs comprising the rest of the somatochart. These reported values were similar to mean somatotype values of for volleyball players [27]. None of the water skiers in this study participated in primarily just one event. Most athletes participated competitively in two or three events. Understanding that the ideal somatotype for a water skier may depend upon the events (slalom, trick, jump) participated in, it is beyond the score of this study to come to conclusions regarding the ideal somatotype for elite performance in each event individually. However, water skiers appear to exhibit a range of somatotypes (Figure 1), comparable to athletes across a number of sports. Ideal somatotype has been shown to change with positional tendency in soccer [7], basketball [25], and gymnastics [5]. Therefore, it is reasonable to surmise that the ideal somatotype for a water skier may differ slightly between events participated in and that higher values for a given component may be more suitable for performance success in a certain water ski discipline over another.

The battery of physical fitness tests revealed a number of interesting findings. Normative running economy data for male and female runners of varying ability levels showed VO_2 max_ values for male collegiate water skiers higher than competitive surfers [28] and similar to highly trained male runners (45.0 mL/kg/min) [29]. Female collegiate women water skiers were comparable to recreational female runners (37.7 mL/kg/min). Mean 40-yard dash times were comparable to regularly active collegiate males and females (5.19 ± 0.23 s and 6.11 ± 0.32 s), respectively [30]. Mean *t*-test times compared favorably to other college athletes, who recorded *t*-test times of 9.94 ± 0.50 s and 10.94 ± 0.60 s [31]. Vertical jump scores also compared favorably to scores from other collegiate athletes, who recorded vertical jump distances of 63.34 ± 11.17 cm and 46.30 ± 8.74 cm, between males and females, respectively [31]. Indicating high levels of physical fitness in collegiate water skiers compared to other collegiate athletes [31]. A significant difference between Y-balance score between men and women for the right and left could not be found (*p* = 0.344 and *p* = 0.147), respectively. Mean sit and reach test scores compared favorably to male and female collegiate distance runners, who exhibited sit and reach scores of 18.38 ± 5.65 cm and 35.88 ± 4.33 cm, respectively [32]. Testing throughout the current study consistently indicated males to exhibit significantly higher scores than females. However, sit and reach test results were contrary, with findings corroborating a previous study showing women were consistently more flexible than men of the same age [33]. The physical fitness tests used in the battery of testing in the study were identified as important to a water skier through previous research [11,12,13]. Whilst water skiing is primarily an anaerobic sport, high levels of aerobic endurance may be beneficial to attenuate the effects of training fatigue from repeated anaerobic activity. Competitive water skiers are required to exhibit high levels of overall physical fitness, dynamic balance, core strength. and flexibility. These physical fitness factors are important to a water skier due to the differing requirements of the three events, the importance of maintaining one’s center of mass on the ski, the unstable surface participated on, and all the alternative situations that can arise from varying external factors.

### 4.2. Potential Morphological and Physical Fitness Factors for Success in Slalom, Trick, Jump Events

A regression analysis indicates that mesomorphic component was associated with slalom score. When seeking to understand why the study found such, it is important to understand the changing nature of the sport over the last 30 years. Undoubtedly the modern-day slalom skier still has to be strong, but arguably does not experience the degree of excess upper body movement short-line slalom skiers used to, through running the slalom course at short line lengths. Previously, the antiquated skiing equipment, and less-advanced, manually driven, towboats, meant it was much harder for a skier to receive a consistent pull. This often led to skiers finding themselves in a less than optimal position throughout a slalom pass. Thus, requiring athletes to often ‘muscle’ their way through a pass and work much harder to hold their direction across the course. Previously, dynamic seated rows (upper back values) for professional water skiers (slalom) were high, compared to the average person [13]. This was understood to be due to the pulling action for the exercise, being similar to the pulling action during water skiing. Upper back strength was previously been shown to be approximately 50% greater than chest press strength in water skiers [12]. Thus, the imbalance of upper body strength appears to be a specific training response inherent to slalom water skiing. The modern sport utilizes advanced towboats, with smarter engines and GPS controlled cruise control, alongside carbon composite skis and advanced binding options, which has unquestionably led to current athletes adopting a smarter approach, with emphasis on technique and precision. Whilst it is still critical for a slalom skier to be strong, and have the musculature necessary to withstand the forces necessary to run short-line slalom passes, the modern-day elite slalom skier is required to be more technique and precision focused, utilizing the power of the boat and the timing of the engine response to the skiers lean more efficiently. This may have led to changing physiological requirements for the modern-day, elite, slalom skier.

The modern approach requires skiers to minimize upper body movement and generate significant angle from the apex of the turn, with an aligned body position, as they move across the wakes, to their decelerating edge (inside edge). The emphasis being placed on controlling unnecessary movement and utilizing force production efficiently may at least in part, explain why the modern-day elite slalom skiers’ somatotype may benefit from lower mesomorphic component.

A regression analysis revealed left lateral flexion time, in seconds, was associated with jump score. A possible explanation for this finding may be found through thinking about sport specific training responses and adaptations that occur due to repetitive exposure to a certain stimulus, in this case, cutting towards the ramp. In the jump event, the athlete will make a counter cut, to help generate width and speed, before making a cut towards the ramp in a balanced position, holding their direction across the course and accelerating though contact with the ramp. The cut to the ramp places considerable strain on the athlete, with sustained isometric contractions of the quadriceps, hamstrings, gluteus maximus [13], allowing the skier to lower their center of mass in the cut and generate speed into the ramp. The latissimus dorsi, erector spinae, internal and external obliques, rotator cuff, biceps, and forearm flexors connect the skier to the rope, and boat. These muscle groups are integral for the skier to maintain proper form through the cut, through strong and sustained isometric contractions. To excel in the left-lateral flexion test requires sustained isometric contractions of the latissimus dorsi, internal and external obliques, and rectus abdominis, alongside other smaller muscles, similarly to the musculature requirements of a water ski jumper in their cut to the ramp.

A regression analysis between trick (hands) performance and selected factors revealed that difference in collegiate trick performance was accounted for by sum of hand grip in 95% of cases studied. Usually the trick event is comprised of two 20-s passes, where an advanced skier will perform two passes, one inclusive of spins, flips, and body-over line tricks, and the other inclusive of toe-hold turns. The trick event collegiately only requires one 20-s pass, where a skier can elect to either do their handle or toe-hold pass. Evidently hand-grip strength is not required during a rope-on-toe pass, so trick analyses were divided into two groups, based on if an athlete used their hands or toe pass in their official NCWSA rankings score. When seeking to understand why hand-grip strength is critical to a trick skier, it is important to understand the requirements of the athlete to be able to use their strength and grip to create load in the line and advance upon the boat to perform the complex higher scoring tricks. A strong grip is critical to maintain control through passing the handle and holding onto big pulls as the skier moves from trick to trick, maintaining their connection to the boat and control of the tension in the line. Whilst there is little value in making recommendations regarding the ideal somatotype and physical fitness components for a trick skier for both passes individually, as all high-end collegiate skiers train both passes for national and international competition, it is worth nothing that mesomorphic component and hand-grip strength appear to be indicators for success in the trick event.

To the best of our knowledge, this study is the first study that examined the morphological and physical fitness profile of water skiers and identified potential morphological and physical factors that are associated with water skiing performance of the slalom, trick, and jump events. However, there are some limitations that need to be considered in the study. First, small sample size may limit generating the valid regression model and the identified factors do not directly lead to water ski success but could potentially be advantageous for a skier. A convenience sampling also can create sampling errors. However, our study utilized highly qualified collegiate athletes who ranked amongst the best in the nation in each event. We also believe that this study would provide important information for designing future studies. Secondly, learning factors such as individual practice time and previous experience may influence the results of water ski performance. In future studies, well-structured study design with large samples size may be required to create the valid regression model for success in slalom, trick, and jump events.

## 5. Conclusions

This study reveals that male water skiers were lower in endomorphic component and better in power, speed, and cardiorespiratory fitness than female water skiers. Morphological factor including a mesomorphic component was negatively associated with slalom event whereas fitness factor such as a left lateral flexion was positively associated with jump event. Most importantly, the sum of hand-grip strength was strongly associated with the trick event score.

## 6. Practical Implication

Physical conditioning plays an important role in performance improvement. This study provides basic information about morphological and physical fitness factors in collegiate water skiers with reference to USA National Championship winning Water Ski Team. Particularly, coaches and athletes may be able to understand key factors that are associated with water ski performance in slalom, trick, and jump events. The battery of physical fitness tests could be utilized by coaches, clinicians, and others interested in identifying an athletes’ strengths and weaknesses, selecting appropriate training regimens, and identifying key physical and physiological features that are attributable to performance success in a water skier.

## Figures and Tables

**Table 1:** Anthropometric and somatotype of collegiate water skiers (Mean ± Standard Deviation).

Variables		Male	Female	t-Score	*p*-Value	ES
Age (years)		20.2 ± 1.93	20.7 ± 3.02			
Body weight (kg)		74.2 ± 8.10 (68.7–81.3)	62.5 ± 8.20 (56.6–68.4)	3.217	0.005	1.43
Height (cm)		178.9 ±7.00 (173.9–183.9)	164.0 ± 5.66 (160.0–168.0)	5.246	0.000	2.34
Body fat percentage (%)		7.7 ± 2.81 (5.7–9.7)	15.1 ± 3.71 (12.5–17.8)	5.084	0.000	2.25
Sum of skinfold thickness (mm)		42.7 ± 14.89 (32.1–53.5)	75.9 ± 17.38 (63.4–88.3)	4.580	0.000	2.05
Somatotype	Endomorphic C.	2.4 ± 0.81 (1.7–3.1)	4.4 ± 1.06 (3.6–5.2)	4.694	0.000	2.12
	Mesomorphic C.	4.3 ± 1.22 (3.2–5.2)	4.1 ± 0.85 (3.5–4.7)	0.321	0.752	0.19
	Ectomorphic C.	2.6 ± 0.97 (1.9–3.4)	1.8 ± 1.10 (1.0–2.5)	1.892	0.075	0.77

Note: Number in parentheses indicates a 95% confidence interval, C = component; ES = effect size.

**Table 2:** Physical fitness in collegiate water skiers (M ± SD).

Fitness Components	Variables	Male (N = 10)	Female (N = 10)	t-Score	*p*-Value	ES
Flexibility	Sit and reach (cm)	71.2 ± 21.83 (57.6–90.3)	90.0 ± 18.13 (77.1–103.0)	2.094	0.051	0.94
Balance	R. Y-balance (score)	82.8 ± 10.55 (73.6–90.5)	79.2 ± 4.79 (75.8–82.6)	0.972	0.344	0.34
	L. Y-balance (score)	86.4 ± 12.97 (75.2–95.9)	79.9 ± 3.80 (77.2–82.6)	1.517	0.159	0.68
Explosive power	Vertical jump (cm)	64.6 ± 8.22 (58.6–71.7)	50.5 ± 6.41 (46.0–55.1)	4.276	0.000	1.91
Speed	40-yard dash (s)	5.1 ± 0.20 (5.0–5.3)	5.8 ± 0.35 (5.6–6.1)	5.086	0.000	2.46
Agility	*t*-test (s)	9.5 ± 0.81 (8.9–10.2)	11.0 ± 1.04 (10.2–11.7)	3.378	0.004	1.61
Muscular strength	R. grip strength (Nm)	35.7 ± 8.82 (30.1–43.4)	31.2 ± 7.05 (26.2–36.2)	1.260	0.224	0.56
	L. grip strength (Nm)	38.3 ± 9.78 (31.8–46.8)	30.6 ± 8.12 (24.8–36.4)	1.915	0.071	0.86
Muscular endurance	R. grip endurance (s)	24.4 ± 6.43 (18.9–29.1)	23.2 ± 12.06 (14.5–31.8)	0.292	0.774	0.12
	L. grip endurance (s)	24.7 ± 8.19 (17.7 -28.5)	20.5 ± 8.66 (14.4–26.7)	1.091	0.290	0.50
	Extension (s)	156.1 ± 44.25 (126.4–194.8)	127.2 ± 39.12 (99.2–155.2)	1.547	0.139	0.69
	R. lateral flexion (s)	108.2 ± 36.64 (79.8–139.1)	92.4 ± 23.75 (76.5–116.47)	0.803	0.432	0.51
	L. lateral flexion (s)	108.8 ± 23.29 (92.3–129.0)	92.4 ± 23.75 (75.4–109.4)	1.559	0.136	0.70
	Push-up (n)	44.8 ± 22.71 (26.9–63.8)	34.3 ± 15.59 (23.2–45.5)	1.206	0.244	0.54
	Body weight squat (n)	62.4 ± 7.11 (22.7–35.6)	61.8 ± 10.29 (54.4–69.2)	0.152	0.881	0.07
Cardiorespiratory fitness	VO_2_max (mL/kg/min)	45.9 ± 9.28 (39.1–53.9)	37.7 ± 5.18 (34.0–41.4)	2.426	0.029	1.01

Note: Number in parentheses indicates 95% confidence interval. R., right; L., left.

**Table 3:** The relationship between and physical fitness and water ski performance.

Variables	Slalom Score	Trick Toes Score	Trick Hands Score	Jump Score
Morphological Factor				
Endomorph component	−0.16	−0.54	−0.10	0.39
Mesomorph component	−0.48 *	0.83 *	−0.32	0.44
Ectomorph component	0.06	−0.03	0.06	−0.60 *
Physical Fitness Factor				
Sit and reach (cm)	−0.30	0.74	0.851	0.30
R. Y-balance (score)	0.34	0.63	−0.68	0.57
L. Y-balance (score)	0.25	0.68	−0.77	0.32
Vertical jump (cm)	0.06	0.45	−0.36	0.30
40-yard dash (s)	−0.06	0.15	0.85	0.37
*t*-test (sec)	−0.17	−0.30	−0.82	0.10
Sum of grip strength (Nm)	−0.12	−0.62	0.98 *	0.11
Sum of grip endurance (s)	0.11	−0.11	0.19	−0.43
Posterior extension (s)	0.12	−0.40	−0.86	0.59 *
Right lateral flexion (s)	−0.05	−0.15	−0.22	0.35
Left lateral flexion (s)	−0.09	−0.71	−0.19	0.63 *
Push-up (n)	0.19	0.60	−0.25	0.45
Body weight squat (n)	0.22	0.56	−0.67	0.51
VO_2_max	0.07	0.51	−0.82	0.02

* *p* < 0.05 indicates significant relations between variables.
